# Temperature dependence of viscosity, relaxation times (T_1_, T_2_) and simulated contrast for potential perfusates in post-mortem MR angiography (PMMRA)

**DOI:** 10.1007/s00414-016-1482-5

**Published:** 2016-11-29

**Authors:** Bridgette Webb, Thomas Widek, Bernhard Neumayer, Christine Bruguier, Sylvia Scheicher, Hanna Sprenger, Silke Grabherr, Thorsten Schwark, Rudolf Stollberger

**Affiliations:** 1 Ludwig Boltzmann Institute for Clinical Forensic Imaging, Universitätsplatz 4/2., Graz, 8010 Austria; 2 Institute of Forensic Medicine, Medical University Graz, Graz, Austria; 3 BioTechMed-Graz, Graz, Austria; 4 University Center of Legal Medicine, Lausanne-Geneva, University of Lausanne, Lausanne, Switzerland; 5 Institute of Medical Engineering, Graz University of Technology, Graz, Austria

**Keywords:** Post-mortem, Temperature, MRI, Quantitative, Simulation, Angiography

## Abstract

Developments in post-mortem imaging increasingly focus on addressing recognised diagnostic weaknesses, especially with regard to suspected natural deaths. Post-mortem MR angiography (PMMRA) may offer additional diagnostic information to help address such weaknesses, specifically in the context of sudden cardiac death. Complete filling of the coronary arteries and acceptable contrast with surrounding tissue are essential for a successful approach to PMMRA. In this work, the suitability of different liquids for inclusion in a targeted PMMRA protocol was evaluated. Factors influencing cooling of paraffinum liquidum + Angiofil® (6 %) in cadavers during routine multiphase post-mortem CT angiography were investigated. The temperature dependence of dynamic viscosity (8–20 °C), longitudinal (T_1_) and transverse (T_2_) relaxation (1–23 °C) of the proposed liquids was quadratically modelled. The relaxation behaviour of these liquids and MR scan parameters were further investigated by simulation of a radiofrequency (RF)-spoiled gradient echo (GRE) sequence to estimate potentially achievable contrast between liquids and post-mortem tissue at different temperatures across a forensically relevant temperature range. Analysis of the established models and simulations indicated that based on dynamic viscosity (27–33 mPa · s), short T_1_ relaxation times (155–207 ms) and a minimal temperature dependence over the investigated range of these parameters, paraffin oil and a solution of paraffin oil + Angiofil® (6 %) would be most suitable for post-mortem reperfusion and examination in MRI.

**Electronic supplementary material:**

The online version of this article (doi:10.1007/s00414-016-1482-5) contains supplementary material, which is available to authorized users.

## Introduction

Since gaining recognition in post-mortem practice in the early 1990s [1], the role and contribution of post-mortem imaging techniques in the investigation of cause of death continue to be extensively evaluated and the concept of minimally invasive autopsies (MIA) continues to gain importance. The usefulness of both post-mortem CT (PMCT), its extension post-mortem CT angiography (PMCTA) and post-mortem MRI (PMMR) as adjuncts in the context of forensic autopsies has been established, and these techniques are increasingly being implemented around the world [2, 3]. PMCT clearly visualises the skeletal system, whilst PMCTA, introduced approximately 10 years ago, delivers an ideal visualisation of the vascular system [4–8]. Finally, PMMR offers excellent soft-tissue contrast which would otherwise be unachievable. In recent years, developments in post-mortem imaging have increasingly focused on addressing recognised diagnostic weaknesses, especially with respect to suspected natural deaths. More specifically, the need to define the role of post-mortem imaging in the assessment of sudden cardiac death (SCD) was underlined in a review of the current state of post-mortem imaging regarding cardiovascular pathologies [9]. The post-mortem evaluation of ischemic heart disease (IHD), the most common underlying cause of SCD, involves examination of the coronary arteries, for stenosis and occlusions as well as examination of the myocardium for signs of ischemia [10]. A combined MIA protocol (CT, MRI and biopsy) used to detect cardiac causes of death was compared to conventional autopsy (CA), resulting in the conclusion that MIA is still insufficient in this area [11]. The need to improve upon this insufficiency currently fuels the development of post-mortem imaging techniques. For example, PMCTA successfully visualises the morphology of coronary arteries to rule out significant stenosis and identify the presence of occlusions [12], whilst PMMR enhances the ability to visualise contrast in soft tissue, with promising performance in the detection of myocardial infarction [13–16]. However, challenges related to infrastructure, the complexity of PMMR (e.g. temperature-dependent contrast) and the influence of post-mortem changes have led to a slower advancement of this technique [4].

Most recently, initial experience has been gained with post-mortem MR angiography (PMMRA) [4]. The technical feasibility of PMMRA was demonstrated using a small cohort; however, sedimentation problems negatively affected image quality [4, 17]. A PMMRA acquisition protocol for ex situ hearts using a lipophilic contrast agent mixture (paraffin oil and Angiofil®) can also be found in the literature [18]; however, to our knowledge, a systematic evaluation of potentially suitable perfusates and imaging protocols in the context of PMMRA has not yet been undertaken.

A complete filling of targeted vessels, specifically of the coronary arteries, is required to enable reliable radiological assessment of the vasculature in the heart. Due to the delicate state and increased permeability of the vascular wall in cadavers, careful consideration of the factors affecting the behaviour of liquids in the vascular system (e.g. nature, viscosity) [4] is required. In current PMCTA applications, aqueous, hygroscopic and lipophilic solutions can be found; however, hygroscopic and lipophilic liquids are often recommended due to their reduced extravasation over time into surrounding tissue [4]. For polyethylene glycol (e.g. PEG200, hygroscopic) solutions, increased viscosity (∼55 mPa · s measured at 20 °C) was found to positively influence the clarity of the vascular image [19]. For paraffin oil (lipophilic), a viscosity of approximately 31 mPa · s (measured at “room temperature”) was considered appropriate, due to its non-observation in capillaries [20]. Furthermore, the viscosity of a given liquid is strongly dependent on its temperature [21]. This was confirmed by examination of different PEG200 solutions, highlighting the need to adapt viscosity to local temperature conditions, including cadaver temperature [22]. A detailed characterisation of the temperature dependence of multiple perfusate viscosities is nevertheless lacking in the literature, meaning that an accurate knowledge of in-cadaver viscosity of liquids used in PMCTA and potentially of interest for PMMRA is not currently available. This study responds to this deficiency and additionally seeks to model dynamic viscosity of potential perfusates over a forensically relevant temperature range.

In addition to a complete filling of the relevant region of the vascular system, knowledge of the attainable contrast in MR images is also necessary when evaluating liquids as potential PMMRA perfusates. Such contrast is determined by the intrinsic properties (e.g., relaxation times) of the administered perfusate and surrounding tissue, as well as by the imaging sequence applied. Studies examining post-mortem tissue and phantoms have already demonstrated that relaxation times are temperature-dependent [23–28], and additional studies implementing PMMR have highlighted the importance of this dependence [13, 16]. The current study provides fundamental information regarding the temperature dependence of the relaxation times (T_1_, T_2_) for a number of potential perfusates. Furthermore, it is possible to approximate the contrast attainable for a selected MR imaging sequence using numerical simulations. To better understand the complex behaviour of different substances in MRI, simulations repeatedly solve the Bloch equations [29] under ideal conditions. Such simulations exploit information intrinsic to the investigated substances, as well as sequence parameters, and are important tools in the development and optimisation of MRI protocols, prior to acquiring experimental MR images.

The main objective of this study was to evaluate the suitability of different liquids for inclusion in a targeted PMMRA protocol. To effectively evaluate such liquids, this work sought to investigate temperature variations for a paraffinum liquidum + Angiofil® (6 %) solution in perfused cadavers, to characterise selected liquids in terms of their temperature-dependent dynamic viscosity and intrinsic MR properties (T_1_, T_2_) and finally to simulate possible contrast achievable against post-mortem tissue using experimentally obtained relaxation times (perfusates) and literature values (cadaveric tissue [27]) using a radiofrequency (RF)-spoiled gradient echo (GRE) sequence.

## Methods

### Influence of cadaver temperature and time on perfusate temperature during CT scans

As PMMRA is not yet performed routinely, the investigation of the influence of cadaver temperature and time on perfusate temperature was performed in reperfused cadavers during routine multiphase post-mortem CT angiography (MPMCTA). The study population consisted of forensic cadavers (*n* = 21) aged between 21 and 75 years at the time of death (mean ± SD: 57.1 ± 14.9; 9 females and 12 males). All cadavers were examined within a post-mortem interval of 3 days or less and underwent a forensic autopsy following MPMCTA. Temperature measurements were conducted on forensic cadavers (both prior to and following routine MPMCTA, performed according to the standardised protocol described by Grabherr et al. [30] (femoral vessels access, 3 phases, injection using Virtangio® perfusion device, 3710 ml of perfusion solution (3500 ml paraffinum liquidum + 210 ml Angiofil® (6 %) mixture)). Of specific interest were cadaver (rectal) temperature at the time of the external examination (ϑ_EE_), temperature of the standardised perfusate (paraffinum liquidum + Angiofil® (6 %)) prior to CT acquisition (ϑ_P0_) and the temperature of the perfusate excreted during the third dynamic phase of MPMCTA (ϑ_P1_, measured in the venous cannula). Furthermore, the total time the excreted perfusate spent in the cadaver (time_C_) was also recorded. All cadavers were scanned within 3 h of the external examination (median: 100 min, range: 26–177 min). The standardised perfusate was stored at ambient temperature (20–24 °C) in the CT scan suite. ANOVA was performed to investigate factors influencing changes in the temperature of the perfusate. Data were fitted to model the influence of cadaver temperature on excreted perfusate temperature (ϑ_P1_) during MPMCTA. The resulting model was evaluated by examining the residual standard error (RSE).

### Characterisation of perfusates and modelling of temperature dependence

Dynamic viscosity and intrinsic MR properties (longitudinal (T_1_) and transverse (T_2_) relaxation times) of liquids considered potentially suitable for targeted cadaveric perfusion (*n* = 9) were characterised across a temperature range of 0.6 to 23.2 °C, which reflects forensically relevant temperatures.

#### Selected perfusates

Lipophilic and hygroscopic solutions, including various hydrocarbons (*n* = 3), polyethylene glycol (*n* = 2), polydimethylsiloxane (silicon oil) as well as paraffin oil, Angiofil®, a contrast agent used in PMCTA, and a paraffin oil + Angiofil® (6 %) solution currently used in forensic practice were experimentally investigated in this study (Online Resource 1_Table 1).

Additionally, a hydrophilic solution (Gadovist® doped water, (2 mmol/l)) was numerically investigated using temperature dependencies established in previous works (dynamic viscosity of water [31], water (T_1_, T_2_) [23] and Gadovist® relaxivity [23]). These data were used to complement experimentally obtained data.

#### Viscosity

Kinematic viscosity and density were measured with an Ubbelohde viscometer (Schott AG, Germany) and density meter (DMA 48, Anton Paar GmbH, Austria) at 8, 10 and 20 °C. Using density values, the obtained kinematic viscosities were converted to dynamic viscosity using Eq. 1 [32]

1 $$ \upmu =\upnu \uprho, $$

where μ is the dynamic (absolute) viscosity (mPa · s), ν the kinematic viscosity (mm^2^/s) and ρ the density (g/cm^3^). Values obtained at 20 °C were used to verify supplier values. For Gadovist® doped water, water values for dynamic viscosity were used [31], as the influence of such a low concentration (2 mmol/l) of Gadovist® was not expected to significantly influence the dynamic viscosity of water.

The temperature dependence of the quantified viscosities was described by the empirical quadratic model in Eq. 2 for temperatures between 8–20 °C.

2 $$ \upmu \left(\upvartheta \right)=\upmu \left(20{}^{\circ}\mathrm{C}\right)+{A}_{visc}\cdot \Delta \upvartheta +{B}_{visc}\cdot \Delta {\upvartheta}^2 $$

Where dynamic viscosity (μ) at a given temperature (ϑ) can be calculated from the dynamic viscosity measured at 20 °C (μ (20 °C)), the difference in temperature (Δϑ = 20-ϑ°C) and two coefficients (A_visc_ and B_visc_), which were defined for each potential perfusate. RSE was used to evaluate the suitability of the quadratic models in describing the temperature dependence of dynamic viscosity for the investigated liquids.

#### Quantitative MRI

MRI measurements were performed on the liquids in polypropylene test tubes on a clinical 3T scanner (Skyra, Siemens AG, Germany) using a 20-channel head/neck coil (Siemens AG, Germany) at four temperatures (1.4, 8.6, 16.1 and 23.2 °C for TIR and 0.6, 8.4, 16.1, 23.2 °C for MSE measurements) (Online Resource 1_Table 2). Inversion recovery (TIR; TR/TE/TI: 12000/9.7/50, 100, 200, 350, 600, 1000, 4000 ms, ETL: 8, slices: 10) and multi-echo SE (MSE; TR: 4000/echo spacing: 10.6 & 20 ms, ETL: 32, slices: 6) sequences were used to acquire quantitative MRI data. Sample temperature was controlled using a water bath and monitored via a real-time fibre optic temperature sensor (Fluoroptic ®, LumaSense Technologies Inc, USA). Between measurements at different temperatures, samples were given sufficient time to adjust.

#### Data analysis

Regions of interest (ROI) corresponding to each liquid were segmented manually per slice (Online Resource 1_Fig. 1). Signal intensity data were fitted using a slice-wise, non-linear least squares, average-then-fit approach for each ROI (270 voxels; n_T1,slices_ = 10, n_T2,slices_ = 6). Analyses were performed in R [33] using the NLME package [34] *nlslist* function. Mono-exponential behaviour was assumed for T_1_ recovery using [35]

3 $$ \mathrm{S}\left(\mathrm{T}\mathrm{I}\right)={\mathrm{S}}_0\left({1\hbox{-} 2\mathrm{A}\mathrm{e}}^{\left(\hbox{-} \frac{\mathrm{T}\mathrm{I}}{{\mathrm{T}}_1}\right)}\right), $$

where S (TI) is the signal measured at a given inversion time (TI). The fitted parameters in Eq. 3 correspond to the signal that would be acquired from the equilibrium longitudinal magnetization (S_0_), a correction factor for incomplete inversion approaching one (A) and the longitudinal relaxation time (T_1_). Data can be found in Online Resource 1_Table 3.

T_2_ decay was also assumed to exhibit mono-exponential behaviour and was modelled using Eq. 4, with the first echo discarded as suggested in [36, 37].

4 $$ \mathrm{S}\left(\mathrm{T}\mathrm{E}\right)={\mathrm{S}}_0{\mathrm{e}}^{\left(\hbox{-} \frac{\mathrm{T}\mathrm{E}}{{\mathrm{T}}_2}\right)} $$

S (TE) is the signal measured as a function of echo time (TE), where the fitted parameters correspond to the signal that would be acquired from the equilibrium longitudinal magnetization (S_0_) and the transverse relaxation time (T_2_). Data can be found in Online Resource 1_Table 3.

The temperature dependence of the quantified relaxation times was described by the empirical quadratic model in Eq. 5 for temperatures between 1–23 °C.

5 $$ {\mathrm{T}}_{1,2}\left(\upvartheta \right)={\mathrm{T}}_{1,2}\left(23{}^{\circ}\mathrm{C}\right)+{A_T}_{{}_{1,2}}\cdot \Delta \upvartheta +{B_T}_{{}_{1,2}}\cdot \Delta {\upvartheta}^2, $$

where relaxation times (T_1_, T_2_) at a given temperature (ϑ) can be calculated from known relaxation times at 23 °C (T_1,2_(23 °C)), the difference in temperature (Δϑ = 23-ϑ°C) and two coefficients (${A}_{T_{1,2}}$and ${B}_{T_{1,2}}$), which were defined for each potential perfusate. The RSE was used to meaningfully evaluate the suitability of the constructed models to describe the temperature dependence of relaxation parameters.

### Evaluation of perfusates for future post-mortem applications

#### Fundamental properties

Liquids were assessed based on fundamental properties such as their nature, dynamic viscosity and relaxation times. Sample T_1_ and T_2_ values, as well as their corresponding temperature dependency models were compared with literature-based relaxation models for cadaveric myocardium and subcutaneous fat (s.c. fat) [27] to identify fundamental differences between liquids and these tissues.

#### Simulations

Bloch equation simulations of a RF-spoiled GRE sequence were performed for selected perfusates, as well as cadaveric tissue, at each of the four experimental temperatures (TE: 5 ms, TR: 20 ms, flip angle: integers between 0–90°, number of simulated spins: 100, number of excitations: 100 and RF phase increment: 117°). An equal proton density contribution was assumed for all simulations. Potential contrast between each perfusate and tissue type was calculated using Eq. 6.

6 $$ {\mathrm{C}}_{\upvartheta}{{}_{,}}_{\mathrm{perfusate}\hbox{-} \mathrm{tissue}}={\mathrm{S}}_{\upvartheta}{{}_{,}}_{\mathrm{perfusate}}-{\mathrm{S}}_{\upvartheta}{{}_{,}}_{\mathrm{tissue}}, $$

in which C_ϑ,perfusate-tissue_ corresponds to the contrast between perfusate and tissue and S_ϑ,perfusate_ and S_ϑ,tissue_ to the signal magnitude of a given perfusate/tissue at a given temperature (ϑ). The optimal flip angle and corresponding maximum expected contrast (C_opt_), defined to four significant figures, were determined for selected perfusate/tissue combinations at each of the investigated temperatures. A range of optimal flip angles for each perfusate/tissue combination over the entire temperature range was further defined. The effects of selecting any flip angle for a given perfusate within the defined range of optimal flip angles were quantified by calculation of the relative difference (d_r_%) in contrast (Eq. 7) at each of the four temperatures.

7 $$ {\mathrm{d}}_r=\frac{\left|{C}_{\mathrm{opt}}-{C}_{\min}\right|}{{\overline{x}}_{\mathrm{opt}, \min }}\ast 100 $$

C_min_ corresponds to the minimum contrast attainable using any of the flip angles within the defined range, whilst C_opt_ refers to the maximum contrast expected, by application of the temperature-specific optimal flip angle. x̅_opt,min_ is the absolute value of the arithmetic mean of C_opt_ and C_min_.

## Results

### Influence of cadaver temperature and time on perfusate temperature during CT scans

**Fig. 1:**
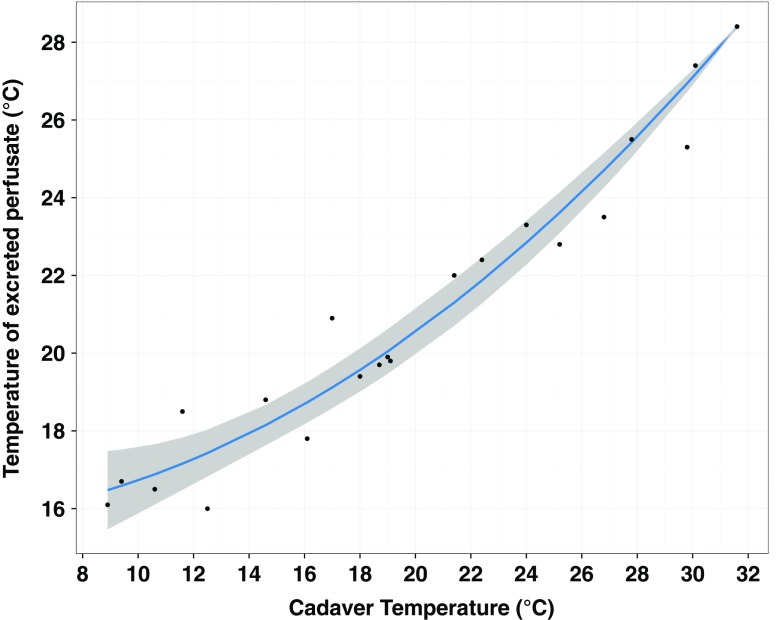
Experimentally measured changes in the temperature of paraffinum liquidum + Angiofil® (6 %) (ϑ_P1_) with respect to cadaver temperature (ϑ_EE_) and corresponding temperature-dependent model according to Eq. 8. *Grey area* indicates the 95 % confidence interval. Data obtained from routine MPMCTA examinations

The measured temperatures and times are presented in Online Resource 1_Table 4. Most notably, there was a strong correlation (Pearson’s *r* = 0.966) between cadaver temperature (ϑ_EE_) and changes in perfusate temperature over the course of routine MPMCTA, represented by the excreted perfusate temperature ϑ_P1_. ANOVA only confirmed the statistical significance of this correlation (Pr < < 0.05), whilst other investigated factors (ϑ_P0,_ time_C_) were not found to be statistically significant (*p* value > > 0.05). A quadratic model was suitable for describing the data (RSE = 0.8778) (Fig. 1). The standard error (SE) of model coefficients was 0.06 and 0.003 for the linear and quadratic terms, respectively.

8 $$ {\upvartheta}_{P1}=28.4+0.835\cdot \Delta {\upvartheta}_{EE}+0.014\cdot \Delta {\upvartheta_{EE}}^2 $$

The term 28.4 corresponds to the post-scan temperature (°C) of the perfusate when ϑ_EE_ was the highest (31.6 °C). Δϑ_EE_ represents all deviations from this temperature and was calculated as 31.6 − ϑ_EE_ (°C). This model is valid for the data range displayed in Fig. 1.

### Characterisation of perfusates and modelling of temperature dependence

#### Viscosity

**Fig. 2:**
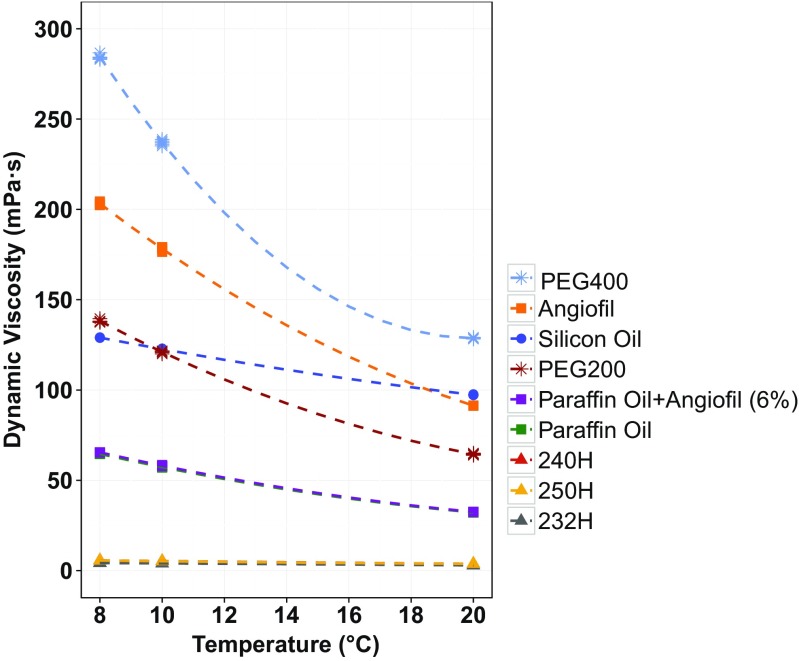
Experimentally measured dynamic viscosities (mPa · s) of potential perfusates at 8, 10 and 20 °C and corresponding temperature dependence models according to Eq. 2. For explanation of abbreviations, see Table 1

Perfusates were classified into three groups based on dynamic viscosity (low: <6, medium: 32–65, high: 65–285 mPa · s) (Online Resource 1_Table 5). The dynamic viscosities of liquids measured at 20 °C were in general agreement with the supplier data (Table 1 and Online Resource 1_Table 1). The highly temperature-dependent nature of dynamic viscosity (negative correlation) was experimentally confirmed and modelled for all samples (Table 1, Fig. 2). The resulting quadratic models produced small RSE (<1.2 mPa · s, Table 1). Polyethylene glycol (PEG) and Angiofil® samples demonstrated the highest temperature dependence.

**Table 1:** Reference dynamic viscosity (μ, at 20 °C), coefficients (A_visc_, B_visc_) for modelled temperature dependence of dynamic viscosity (mPa · s) according to Eq. 2 and the residual standard error (mPa · s) for each model

Perfusate	μ (20 °C)	A_visc_	B_visc_	RSE
232 Hydroseal® (232H)	2.9	−0.11	−0.0003	0.0055
240 Hydroseal® (240H)	3.7	−0.14	0.002	0.0084
250 Hydroseal® (250H)	3.7	−0.10	0.005	0.0071
PEG200	64.5	−3.22	0.243	0.54
PEG400	128.7	−0.11	1.072	1.16
Silicon oil	97.3	−2.00	0.053	0.16
Paraffin oil	32.2	−1.54	0.097	0.11
Angiofil®	91.4	−5.51	0.318	0.86
Paraffin oil + Angiofil® (6 %)	32.5	−1.62	0.094	0.15

#### Quantitative MRI

**Fig. 3:**
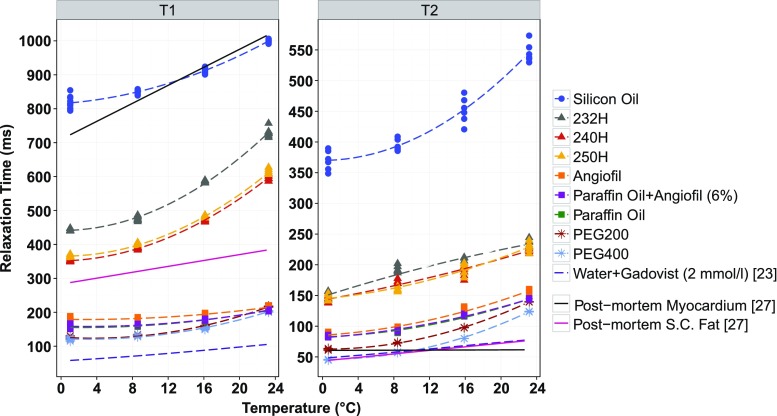
Calculated relaxation times (T_1_, T_2_) from experimental measurements of all investigated liquids at 1, 8.5, 16 and 23 °C with their corresponding temperature dependence models according to Eq. 5. For comparison, numerically investigated cadaveric tissue and Gadovist® doped water (2 mmol/l) based on values and models in [23, 27] are also displayed. For explanation of abbreviations, see Table 1

Temperature measurements were stable over the duration of each scan session with the standard deviation of measurements being less than 0.5 °C for all temperatures except 23.2 °C, where it was 1.6 and 0.9 °C for TIR and MSE measurements, respectively (Online Resource 1_Table 2). Standard deviation of the calculated T_1_ and T_2_ values was small (relative standard deviation (RSD) < 3.2 %). There was a positive correlation between temperature and relaxation time (T_1_: Pearson’s *r* > 0.912; T_2_: Pearson’s *r* > 0.941). This correlation was stronger for some samples (e.g. Hydroseal®) than for others (e.g. paraffin oil, Angiofil®). Temperature dependence of T_1_ and T_2_ values was reliably modelled using Eq. 5 (Table 2, Fig. 3). The resulting quadratic models displayed small RSE (Table 2).

**Table 2:** Reference relaxation times (T_1_ and T_2_, at 23 °C), coefficients (*AT*_1,2_ *, BT*_1,2_ for modelled temperature dependence of T_1_ and T_2_ (ms) according to Eq. 5 and corresponding residual standard error (ms) for each model

Perfusate	T_1_ (ms)				T_2_ (ms)			
	T_1_ (23 °C)	*AT*_1_	*BT*_1_	RSE	T_2_ (23 °C)	*AT*_2_	*BT*_2_	RSE
232H	729.6	24.8	0.53	7.2	233.6	2.7	−0.04	9.7
240H	597.0	20.6	0.43	4.6	223.0	4.3	0.04	6.5
250H	611.5	21.4	0.46	4.6	229.0	5.9	0.10	7.0
PEG200	216.2	9.5	0.24	3.4	139.4	6.7	0.15	1.0
PEG400	201.9	8.1	0.20	3.2	123.5	6.8	0.15	1.6
Silicon oil	999.4	14.1	0.27	10.7	545.5	15.1	0.32	15.5
Paraffin oil	206.7	4.9	0.12	2.7	144.3	4.8	0.09	2.4
Angiofil®	214.8	3.5	0.08	3.5	157.7	5.4	0.10	4.8
Paraffin oil + Angiofil® (6 %)	205.5	4.7	0.11	2.7	144.1	4.4	0.07	2.4

### Evaluation of perfusates for future post-mortem applications

#### Nature and dynamic viscosity

Hygroscopic, hydrophilic and lipophilic liquids all find applications in current approaches to post-mortem CT angiography [6]. Hygroscopic liquids draw water molecules from the surrounding environment, which is undesirable for an approach to post-mortem MR angiography as it introduces an unknown “water content” factor into the system, meaning that the relaxation behaviour of the perfused liquid becomes less predictable. Therefore, for an initial targeted approach to post-mortem MR angiography, either a lipophilic or hydrophilic liquid was preferred. Liquids demonstrating medium viscosity (i.e. 20–100 mPa · s over the defined temperature range were also preferred to enable reliable vessel filling and acceptable intravascular retention. Considering the physical characteristics (Fig. 2), preferred liquids were paraffin oil and the paraffin oil + Angiofil® (6 %) solution.

#### Relaxation (T1, T2) properties

Differences in the relaxation behaviour (T_1_, T_2_) of potential perfusates and cadaver tissue were identified with the aid of linear and quadratic models over the temperature range (0.6–23.2 °C) (Fig. 3). With the exception of silicon oil, T_1_ relaxation in all potential perfusates was distinctly different to that in cadaveric myocardium. Acceptable positive contrast could therefore be expected between any of these perfusates and myocardium using T_1_-weighted imaging. A similar observation could be made for cadaveric s.c. fat, albeit that the Hydroseal® samples (232H, 240H and 250H) would, in this case, no longer provide a positive contrast against cadaveric s.c. fat due to their longer T_1_. T_2_ relaxation times for cadaveric tissue and potential perfusates were much closer together. Nevertheless, all lipophilic perfusates demonstrated slightly longer transverse relaxation times compared to the investigated tissues, suggesting positive contrast in T_2_-weighted images.

Overall, T_1_ values for PEG, paraffin oil, Angiofil®, paraffin oil + Angiofil® (6 %) and Gadovist® doped water (2 mmol/l) lay at least 100 ms below corresponding cadaveric tissue values (Online Resource 1_Table 3). For all perfusates except PEG and Gadovist® doped water (2 mmol/l), T_2_ values were longer than tissue T_2_ values. Preferred liquids based on relaxation properties were paraffin oil, Angiofil® as well as the paraffin oil + Angiofil® (6 %) solution and Gadovist® doped water (2 mmol/l).

#### Simulations and potential contrast

**Fig. 4:**
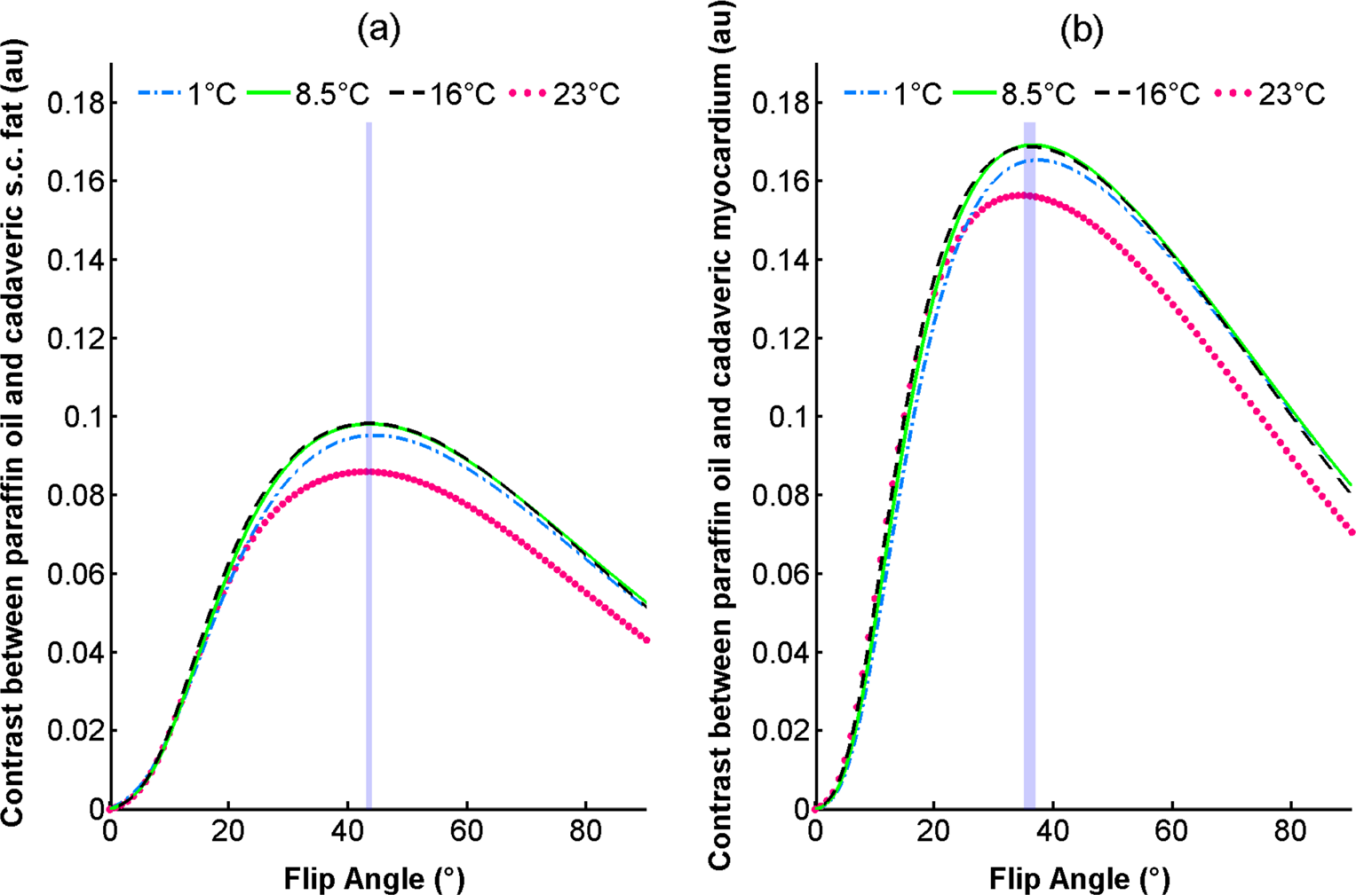
Bloch equation simulation of RF-spoiled GRE sequence. Contrast between paraffin oil and cadaveric s.c. fat (**a**) and between paraffin oil and cadaveric myocardium (**b**) at 1, 8.5, 16 and 23 °C. The *shaded area* in both plots corresponds to the defined range of optimal flip angles (Table 3)

Based on their physical and relaxation properties, only paraffin oil and the paraffin oil + Angiofil® (6 %) solution were considered potentially suitable for targeted cadaver perfusion and examination in MRI. Contrast potentially achievable between suitable perfusates and cadaveric tissue (myocardium/s.c. fat [27]) at 1, 8.5, 16 and 23 °C was investigated by simulating a RF-spoiled GRE sequence. For simplicity, only simulations for paraffin oil are shown; however, both perfusates performed comparably across all temperatures. Overall, simulated contrast between perfusates and s.c. fat (shown for paraffin oil in Fig. 4a) was lower than that between perfusates and myocardium (shown for paraffin oil in Fig. 4b).

At a given temperature, maximum contrast between perfusates and surrounding tissue could be achieved with either one or two optimal flip angles (Online Resource 1_Table 6). The flip angles optimising contrast were found to be smaller for cadaveric myocardium than for s.c. fat. Different optimal flip angles were observed at each of the investigated temperatures, with a general trend towards smaller flip angles at higher temperatures (Online Resource 1_Table 6). The greatest discrepancy was 4° over the entire temperature range. This difference translated into a maximum relative difference (d_r_) in simulated contrast of only 0.50 % (Table 3, paraffin oil + Angiofil® and myocardium) at a given temperature. However, when optimal flip angles were selected and contrast between selected perfusates and cadaveric tissue over the entire temperature range examined, larger relative differences in simulated contrast (d_r_) of up to 13.25 % due to differences in temperature were observed.

**Table 3:** Range of simulated flip angles which maximises contrast between selected perfusates (paraffin oil and a paraffin oil + Angiofil® (6 %) solution) and cadaveric tissue (s.c. fat and myocardium, [27]) for temperatures between 1–23 °C. Maximum relative difference in contrast (d_r_%) at four temperatures resulting from the use of all flip angles in the defined range

Range of optimal flip angles (°)				
Temperature (°C)	Paraffin oil		Paraffin oil + Angiofil® (6 %)	
	s.c. Fat	Myocardium	s.c. Fat	Myocardium
1	43–44	35–37	43–44	34–37
8.5	43–44	35–37	43–44	34–37
16	43–44	35–37	43–44	34–37
23	43–44	35–37	43–44	34–37
Maximum relative difference (d_r_) in contrast (%)				
Temperature (°C)	Paraffin oil		Paraffin oil + Angiofil® (6 %)	
	s.c. Fat	Myocardium	s.c. Fat	Myocardium
1	0.07	0.24	0.07	0.50
8.5	0.01	0.06	0.01	0.30
16	0.01	0.06	0.02	0.18
23	0.03	0.26	0.03	0.19

## Discussion

### Influence of cadaver temperature on in-cadaver temperature of paraffinum liquidum + Angiofil® (6 %)

The correlation between cadaver temperature and the reduced in-cadaver temperature of paraffinum liquidum + Angiofil® (6 %) was significant. The proposed quadratic model explained this correlation well. It was also hypothesised, that the total time the perfusate spent in a cadaver would influence changes in its temperature. However, this factor was not found to be statistically significant in this study (*p* value > > 0.05). Nevertheless, it is important to note that in the current study, the perfusate was only held in the cadaver for a short and similar duration in all cases (max. 23 min). Time may become more significant if the perfusate were to be held for hours in the cadavers (e.g. during long scan times or between CT and MRI scan sessions). Furthermore, if a smaller volume of perfusate were to be used, for example in a targeted post-mortem angiographic approach, a quicker equilibration of perfusate temperature with cadaver temperature would be expected. Finally, despite cadavers having core temperatures as low as 9 °C in some cases, perfusate temperature in the cadaver did not descend below 15 °C. Once again, the short duration may have meant that perfusate did not always have enough time to reach this lower core cadaver temperature.

### Characterisation, temperature-dependent modelling and evaluation of potential perfusates

#### Physical characteristics

A successful, targeted approach to post-mortem MR angiography requires a complete filling of the coronary arteries to enable reliable radiological assessment of the heart vasculature. Due to the delicate state and increased permeability of the vascular wall in cadavers, viscosity and the nature of a liquid (hydrophilic, lipophilic or hygroscopic) both play an important role in controlling intravascular retention as well as the smallest diameter of the vessels able to be filled [4, 22]. A compromise between these conditions was therefore required. The temperature-dependent characterisation of dynamic viscosity formed an essential part of this systematic evaluation of liquids proposed for post-mortem perfusion. To date, the majority of studies in this area have examined this important physical property at 20 or 25 °C [19, 22]. As demonstrated by our investigation of perfusate temperature changes in cadavers, the in-cadaver perfusate temperature can decrease to at least 15 °C during post-mortem imaging, if not lower over an extended period of time.

Combining information gained from the determined quadratic models with the established relationship between dynamic viscosity and vessel filling, liquids which could reliably perfuse all vessels of interest over a forensically relevant temperature range were defined. An in-cadaver dynamic viscosity range between 20–100 mPa · s was deemed appropriate. Viscosities above 15 mPa · s have been shown to prevent capillary distribution of the perfusate [22], whilst higher viscosities (∼55 mPa · s) have indicated a longer intravascular retention [19], up to a certain point. Once the viscosity becomes too high, the perfusate can no longer pass through the small arteriovenous shunts and essential vasculature may not be reliably filled. Furthermore, minimal temperature dependence was preferred to minimise changes in vessel filling due to variations in temperature. Examination of the quadratic models explaining changes in dynamic viscosity due to temperature indicated that, based on small RSE, they were suitable for this purpose. A closer look at the coefficients revealed that the preferred liquids all displayed similar temperature dependence (Table 1, A_visc_ = −1.54–1.62, B_visc_ = 0.094–0.097). This translates to dynamic viscosities of approximately 27–33 mPa · s over the temperature range 8–20 °C.

#### Relaxation (T_1_, T_2_) properties

The intrinsic properties of a substance or tissue lay the basis for signal intensity, and correspondingly contrast, in MRI. Therefore, characterisation and evaluation of these properties in liquids being considered for a post-mortem approach to MR angiography were essential. Liquids were characterised in test tubes at 3T for four temperatures across a forensically relevant temperature range. The ex situ (phantom) approach taken in this study is commonly used in a biomedical engineering context to create somewhat controlled conditions enabling an accurate characterisation of the investigated material. A positive correlation between relaxation times (T_1_, T_2_) and temperature was confirmed and modelled. The quadratic models used to fit the data resulted in small RSE, indicating their suitability for this purpose. Achieving positive contrast between a perfused liquid and surrounding tissue requires liquids to have a shorter longitudinal relaxation time (hyperintense on T_1_-weighted images) and/or a longer transverse relaxation time (hyperintense on T_2_-weighted images), compared with surrounding tissue. Experimentally obtained T_1_ relaxation times in polyethylene glycol, paraffin oil, Angiofil®, paraffin oil + Angiofil® (6 %) and Gadovist® doped water (2 mmol/l) [23] were shorter than values for cadaveric tissue [27], indicating that these liquids would appear brighter than the tissue on T_1_-weighted images. Whilst it is undisputable that Gadovist® doped water would deliver excellent T_1_ contrast, the viscosity is too low to enable reliable post-mortem intravascular retention over a longer period of time. The experimentally obtained T_2_ relaxation times for all perfusates except PEG were longer than in-cadaveric tissue as measured in [27], indicating that these liquids would appear brighter than the tissue on T_2_-weighted images. T_1_ and T_2_ relaxation of the preferred liquids (paraffin oil and the paraffin oil + Angiofil® (6 %) solution) over the investigated temperature range were very similar. Both would be equally suitable perfusates in an approach to post-mortem MR angiography. The largest intrinsic differences between perfusates and the investigated post-mortem tissues, based on values and models in [27] were in the longitudinal relaxation times, indicating T_1_-weighted imaging sequences may be better suited to optimise contrast between such perfusates and surrounding cadaveric tissue in PMMRA.

### Simulations of potential contrast between selected perfusates and cadaveric tissue

Signal simulations are an important tool for predicting contrast between a potential perfusate and cadaveric tissue [27] present in the anatomical region of interest. Current literature lacks reliable values for epicardial fat, which led the authors to use cadaveric myocardium and s.c. fat values [27] for simulations. These tissues and their relaxation properties, previously modelled in [27], were used to estimate potential contrast and define optimal sequence parameters for the selected perfusate. Given the clinical importance of RF-spoiled GRE sequences in MR angiography, simulations of this sequence were performed to evaluate the preferred perfusates and to identify variations in the optimal flip angle, which maximises contrast between a perfused liquid and the surrounding tissue, across the investigated temperature range. Simulation parameters were chosen with a post-mortem application in mind, for example an increased value was used for TR, which would be undesirable in a clinical setting where time restrictions due to first-pass enhancement play a more important role.

Due to the lipophilic nature of the perfusates, a better contrast between perfusates and myocardium than that between perfusates and s.c. fat was expected. Simulations confirmed these expectations (Fig. 4). Although to a lesser extent, positive contrast between preferred perfusates and cadaveric s.c. fat was nevertheless evident in the simulations. Given the novel application of MRI to visualise perfused liquids in cadavers, scan parameters such as flip angle, were also thoroughly investigated. The observed slight decrease in the optimal flip angle with increasing temperature in the simulations can be explained by the T_1_-weighting of the RF-spoiled GRE sequence, due to selected parameters, and the observed temperature dependence of T_1_. Despite this relationship, the relative difference in contrast resulting from the use of an optimal flip angle corresponding to a different temperature between 1 and 23 °C was found to be very small (max. 0.50 %). This small relative difference in contrast indicates that even with an approximate optimal flip angle, contrast could be maximised. Nevertheless, the effects of temperature cannot be completely disregarded. Simulations clearly demonstrated differences in the maximum contrast achievable at different temperatures. Interestingly, contrast at lower temperatures (1, 8.5 and 16 °C) was higher than that at 23 °C. This may be due to the stronger temperature dependence of relaxation properties in s.c. fat and myocardium compared to in the preferred perfusates. Simulations demonstrated that this difference in the temperature dependence of the intrinsic relaxation properties resulted into a more dramatic decrease in tissue signal than in perfusate signal as temperature decreased, effectively leading to an increase in contrast between the preferred perfusates and tissue at lower temperatures.

## Conclusion

This work evaluated the suitability of different liquids for inclusion in a targeted PMMRA protocol. Two preferred liquids, paraffin oil and a solution of paraffin oil + Angiofil® (6 %), were identified. The quadratic model approximating in-cadaver temperature of paraffinum liquidum + Angiofil® (6 %) based on cadaver temperature at the time of external examination may also be applied more generally for other liquids with similar physical properties. The characterised dynamic viscosities and intrinsic MR properties (T_1_, T_2_), as well as their temperature-dependent models, were used to evaluate the suitability of liquids for use as perfusates in a targeted approach to PMMRA, leading to the identification of the two preferred liquids. Simulation of a RF-spoiled GRE sequence revealed the potential contrast achievable between these preferred liquids and relevant cadaveric tissues based on the modelled temperature dependence of relaxation times found in [27]. Interestingly, differences in the temperature dependence of relaxation properties for post-mortem tissues [27] and the preferred perfusates led to better contrast in simulations at lower temperatures.

The approach described in this work provides important information for optimising sequence parameters, especially at institutes where scanner access for forensic cases is limited. The study contributes fundamental knowledge of potential perfusates as well as a preliminary exploration of MR sequencing parameters to aid in the systematic development of PMMRA, with the ultimate goal of further improving minimally invasive post-mortem diagnosis of sudden cardiac death.

## Electronic supplementary material

Below is the link to the electronic supplementary material.

ESM 1 ᅟ(DOCX 94 kb)
