# Supplementary material for: Temperature dependence of viscosity, relaxation times (T1, T2) and simulated contrast for potential perfusates in post-mortem MR angiography (PMMRA)
Source: Int J Legal Med. 2016 Nov 29;131(3):739–49. doi: 10.1007/s00414-016-1482-5 (PMC5388705; doi:10.1007/s00414-016-1482-5)
Supplement: Supplementary file 1 — ᅟ(DOCX 94 kb) [file 414_2016_1482_MOESM1_ESM.docx]

***International Journal of Legal Medicine***

**Temperature-dependence of viscosity, relaxation times (T_1_, T_2_) and simulated contrast for potential perfusates in post-mortem MR angiography (PMMRA)**

Bridgette Webb^1,5^, Thomas Widek^1,5^, Bernhard Neumayer^1,5^, Christine Bruguier^3^, Sylvia Scheicher^1,5^, Hanna Sprenger^1,5^, Silke Grabherr^3^, Thorsten Schwark^1,3^ and Rudolf Stollberger^4,5^

^1^ Ludwig Boltzmann Institute for Clinical Forensic Imaging, Graz, Austria.

^2^ University Center of Legal Medicine, Lausanne-Geneva, University of Lausanne, Switzerland.

^3^ Institute of Forensic Medicine, Medical University Graz, Austria.

^4^ Institute of Medical Engineering, Graz University of Technology, Austria.

^5^ BioTechMed-Graz, Austria.

**CORRESPONDENCE:**

Email: [bridgette.webb@cfi.lbg.ac.at](mailto:bridgette.webb@cfi.lbg.ac.at)

# Supplementary Material (Online resource)

Fig. 1: Example slice of quantification data, colours correspond to the ROIs


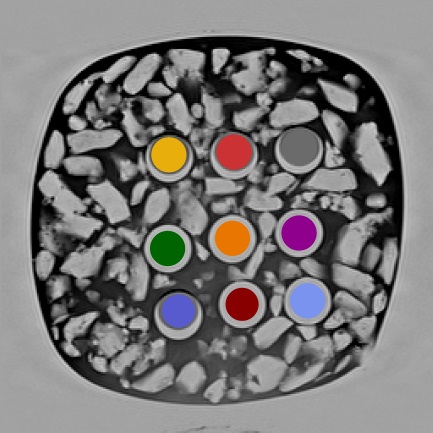


Table 1: Sample nature, description and supplied dynamic viscosity data (mPa·s)

| **Liquid** | **Nature** | **Description** | **Temperature (°C)** | **Supplied Dynamic Viscosity**  **(mPa·s)** |
| --- | --- | --- | --- | --- |
| 232H | Lipophilic | Hydrocarbons (C13-16, <0,03% aromatics) | 20 | 2.9 |
| 240H | Lipophilic | Hydrocarbons (C15-20, <0,03% aromatics) | 20 | 3.7 |
| 250H | Lipophilic |  | 20 | 4.7 |
| PEG200 | Hygroscopic | Polyethylene glycol, a polymer of ethylene oxide. PEGG200 (mol wt: 190-210, PEG400 (mol wt: 380-420). | 20 | 60-67 |
| PEG400 | Hygroscopic |  | 20 | 105-130 |
| Silicon oil | Lipophilic | Liquid polymerized siloxane (polydimethylsiloxane). | 25 | ~ 95 |
| Paraffin oil | Lipophilic | Highly refined mineral oil, n-alkane based | 20 | 28-36 |
| Angiofil® | Lipophilic | Oil-based, non-polar CT contrast component | 20 | 61.3 |
| Paraffin Oil + Angiofil® (6%) | Lipophilic | Mixture of paraffin oil and Angiofil ® (6%) | - | - |
| Water | Hydrophilic | - | 20 | 1.0016 [31] |

Table 2: Mean temperature (°C) of the water-bath for (a) inversion recovery and (b) multi-echo SE sequences

| Mean temperature (SD) (°C) | |
| --- | --- |
| **(a)** | **(b)** |
| 1.4 (0.4) | 0.6 (0.3) |
| 8.6 (0.3) | 8.4 (0.2) |
| 16.1 (0.3) | 16.1 (0.3) |
| 23.2 (1.6) | 23.2 (0.9) |

Table 3: T_1_ and T_2_ relaxation times for investigated perfusates at four temperatures and numerically calculated T_1_ and T_2_ relaxation times for cadaveric myocardium and s.c. fat at the same four temperatures (linear models, [27])

| Perfusate | T_1_ (SD) (ms) | | | | T_2_ (SD) (ms) | | | |
| --- | --- | --- | --- | --- | --- | --- | --- | --- |
|  | **1.4°C** | **8.6°C** | **16.1°C** | **23. 2°C** | **0.6°C** | **8.4°C** | **16.1°C** | **23.2°C** |
| 232H | 443 (4) | 477 (6 | 584 (3) | 730 (11) | 148 (7) | 193 (6) | 203 (9) | 234 (8) |
| 240H | 352 (3) | 389 (3 | 471 (3) | 597 (8) | 143 (5) | 172 (3) | 189 (9) | 223 (4) |
| 250H | 365 (4) | 400 (3 | 481 (3) | 612 (7) | 146 (5) | 160 (4) | 194 (10) | 229 (8) |
| PEG200 | 124 (4) | 133 (2 | 158 (2) | 216 (2) | 62 (1) | 73 (1) | 98 (1) | 139 (1) |
| PEG400 | 121 (4) | 129 (2 | 152 (2) | 202 (2) | 45 (1) | 58 (1) | 80 (1) | 124 (1) |
| Silicon oil | 818 (19) | 849 (7 | 914 (8) | 999 (5) | 370 (16) | 395 (10) | 452 (21) | 546 (16) |
| Paraffin oil | 155 (3) | 161 (2 | 176 (2) | 207 (3) | 84 (1) | 91 (2) | 117 (1) | 144 (1) |
| Angiofil® | 179 (6) | 181 (3 | 195 (3) | 215 (2) | 88 (2) | 94 (4) | 129 (2) | 158 (3) |
| Paraffin oil + Angiofil® (6%) | 158 (4) | 163 (2 | 178 (2) | 206 (2) | 83 (2) | 92 (1) | 119 (1) | 144 (1) |
| **Cadaveric tissue** | **1.4°C** | **8.6°C** | **16.1°C** | **23. 2°C** | **0.6°C** | **8.4°C** | **16.1°C** | **23.2°C** |
| Myocardium [27] | 723.1 | 823.7 | 923.3 | 1017.6 | 60.6 | 60.9 | 61.2 | 61.6 |
| S.c. fat[27] | 289.2 | 320.5 | 353.1 | 384.0 | 44.2 | 55.5 | 66.3 | 76.8 |

**Table 4: Cadaver temperature (°C) at the time of the external examination (ϑ_EE_), temperature prior to perfusion (ϑ_P0_) and following excretion (ϑ_P1_), as well as time measurements (min)**

| **Case** | **ϑ_EE_ (°C)** | **ϑ_P0_ (°C)** | **ϑ_P1_ (°C)** | **Time_C_ (min)** |
| --- | --- | --- | --- | --- |
| 1 | 9.4 | 21 | 16.7 | 15 |
| 2 | 8.9 | 21 | 16.1 | 8 |
| 3 | 12.5 | 21 | 16 | 8 |
| 4 | 30.1 | 20.6 | 27.4 | 7 |
| 5 | 18.7 | 20.6 | 19.7 | 10 |
| 6 | 18 | 20.7 | 19.4 | 13 |
| 7 | 29.8 | 21.6 | 25.3 | 7 |
| 8 | 10.6 | 21.6 | 16.5 | 22 |
| 9 | 19.1 | 21.3 | 19.8 | 9 |
| 10 | 31.6 | 21.2 | 28.4 | 12 |
| 11 | 25.2 | 21.7 | 22.8 | 15 |
| 12 | 21.4 | 21.1 | 22 | 16 |
| 13 | 22.4 | 21 | 22.4 | 10 |
| 14 | 16.1 | 20.9 | 17.8 | 16 |
| 15 | 14.6 | 21 | 18.8 | 14 |
| 16 | 19 | 21.5 | 19.9 | 18 |
| 17 | 26.8 | 21.9 | 23.5 | 23 |
| 18 | 24 | 21.1 | 23.3 | 15 |
| 19 | 17 | 21.8 | 20.9 | 17 |
| 20 | 27.8 | 21.5 | 25.5 | 18 |
| 21 | 11.6 | 21.7 | 18.5 | 11 |

Table 5: Mean dynamic viscosity (mPa·s) and standard deviations (SD) of all perfusates at 8, 10, 20°C.

| Perfusate | Mean dynamic viscosity (SD) (mPa·s) | | |
| --- | --- | --- | --- |
|  | **8°C** | **10°C** | **20°C** |
| **< 6 mPa·s** | | | |
| 232H | 4.221 (0.007) | 4.010 (0.006) | 2.930 (0.004) |
| 240H | 5.62 (0.01) | 5.28 (0.01) | 3.730 (0.002) |
| 250H | 5.62 (0.01) | 5.21 (0.01) | 3.730 (0.003) |
| **32-65 mPa·s** | | | |
| Paraffin oil | 64.6 (0.1) | 57.3 (0.2) | 32.23 (0.04) |
| Paraffin oil + Angiofil® (6%) | 65.5 (0.1) | 58.1 (0.2) | 32.52 (0.06) |
| **65-285 mPa·s** | | | |
| PEG200 | 138.2 (0.8) | 121.1 (0.7) | 64.5 (0.3) |
| Angiofil® | 203.2 (0.8) | 179.2 (1.1) | 91.4 (0.2) |
| Silicon oil | 128.9 (0.1) | 122.7 (0.7) | 97.3 (0.1) |
| PEG400 | 284.3 (1.4) | 237 (1.4) | 128.7 (0.4) |

**Table 6: Flip angles (°) optimising simulated contrast between perfusates (paraffin oil, paraffin oil + Angiofil®) and cadaveric tissue (s.c. fat and myocardium, [27]) at 1, 8.5, 16 and 23°C.**

| Optimal flip angles (°) | | | | |
| --- | --- | --- | --- | --- |
| Temperature (°C) | **Paraffin oil** | | **Paraffin oil + Angiofil ® (6%)** | |
|  | **s.c. fat** | **myocardium** | **s.c. fat** | **myocardium** |
| 1 | 44 | 37, 38 | 44 | 37 |
| 8.5 | 44 | 36, 37 | 44 | 36, 37 |
| 16 | 44 | 36, 37 | 44 | 36, 37 |
| 23 | 43 | 35 | 43 | 34, 35 |
